# A value proposition for early physical therapist management of neck pain: a retrospective cohort analysis

**DOI:** 10.1186/s12913-016-1504-5

**Published:** 2016-07-12

**Authors:** Maggie E. Horn, Gerard P. Brennan, Steven Z. George, Jeffrey S. Harman, Mark D. Bishop

**Affiliations:** Department of Rehabilitation Sciences, University of Oklahoma Health Sciences Center, 1200 N Stonewall Ave, Oklahoma, OK 73117 USA; Director of Clinical Quality and Outcomes Research, Intermountain Healthcare, 389 South 900 East, Salt Lake, UT 84102 USA; Department of Physical Therapy, University of Florida, UFHSC, Box 100154, Gainesville, FL 32610 USA; Department of Behavioral Science and Social Medicine, Florida State University College of Medicine, 1115 West Call Street, Tallahassee, FL 32606 USA

**Keywords:** Value, Efficiency, Neck pain, Pain, Disability

## Abstract

**Background:**

Neck pain is one of the most common reasons for entry into the healthcare system. Recent increases in healthcare utilization and medical costs have not correlated with improvements in health. Therefore there is a need to identify management strategies for neck pain that are effective for the patient, cost efficient for the payer and provided at the optimal time during an episode of neck pain.

**Methods:**

One thousand five hundred thirty-one patients who underwent physical therapist management with a primary complaint of non-specific neck pain from January 1, 2008 to December 31, 2012 were identified from the Rehabilitation Outcomes Management System (ROMS) database at Intermountain Healthcare. Patients reporting duration of symptoms less than 4 weeks were designated as undergoing “early” management and patients with duration of symptoms greater than 4 weeks were designated as receiving “delayed” management. These groups were compared using binary logistic regression to examine odds of achieving Minimal Clinically Important Difference (MCID) on the Neck Disability Index (NDI) and Numerical Pain Rating Scale (NPRS). Separate generalized linear modeling examined the effect of timing of physical therapist management on the metrics of value and efficiency.

**Results:**

Patients who received early physical therapist management had increased odds of achieving MCID on the NDI (aOR = 2.01, 95 % CI 1.57, 2.56) and MCID on the NPRS (aOR = 1.82, 95 % CI 1.42, 2.38), when compared to patients receiving delayed management. Patients who received early management demonstrated the greatest value in decreasing disability with a 2.27 percentage point change in NDI score per 100 dollars, best value in decreasing pain with a 0.38 point change on the NPRS per 100 dollars. Finally, patients receiving early management were managed more efficiently with a 3.44 percentage point change in NDI score per visit and 0.57 point change in NPRS score per visit.

**Conclusions:**

These findings suggest that healthcare systems that provide pathways for patients to receive early physical therapist management of neck pain may realize improved patient outcomes, greater value and higher efficiency in decreasing disability and pain compared to delayed management. Further research is needed to confirm this assertion.

## Background

The majority of healthcare in the United States is delivered in a fee for service (FFS) model and this delivery model has likely contributed to the uncontrollable rise in healthcare costs in the United States [1–3]. Under this system, healthcare providers may be financially incentivized to provide a greater volume of services without regard for improved outcomes, provider performance or quality. In fact, utilization and medical costs for spine conditions have increased under this system but these increases in cost did not correlate with improvements in health [4]. Therefore there is a need to identify and prioritize management strategies that demonstrate high “value proposition” [5, 6] by being effective for the patient, cost efficient for the payer and provided at the optimal time during an episode of pain for enhanced benefit. Indeed, estimating the value proposition of services provided by physical therapists for common musculoskeletal pain conditions has been highlighted as a priority by the profession [5] and in draft documents for the National Pain Report [7].

Recent research supports that early physical therapist management for low back pain is recommended to improve outcomes in comparison to other management pathways such as advanced imaging, prescription medication or advanced care [8, 9], and can lead to lower downstream healthcare utilization and costs [10, 11]. This recommendation differs from previous recommendations where management is delayed to account for potential spontaneous recovery and advised only for patients that are resistant to recovery [12–14]. Despite neck pain being the second most common musculoskeletal disorder after low back pain [15, 16] and physical therapists being the most frequently visited healthcare provider for neck pain [17], the effect of early physical therapist management on outcomes has not been investigated in patients with neck pain.

Neck pain is one of the most common reasons for entry into the healthcare system [18] and disability related to neck pain has an enormous impact on individuals and their families, communities and healthcare systems [19–22]. Therefore there is a need to prioritize management strategies to that improve outcomes and decrease the financial burden of neck pain. To examine the value proposition of early physical therapist management of neck pain, the metrics of value and efficiency for physical therapist management need to be explored. Porter (2010) defines value as the health outcomes achieved per dollar [23]. If value improves, patients, payers, and providers can all benefit while the economic sustainability of the health care system increases [23]. Moreover, the efficiency of delivery of care is important; that is, how quickly does a patient improve? Greater efficiency in managing neck pain may lead to a decrease in indirect costs and allow the provider manage the patient more effectively with less utilization of resources.

The purpose of the study was, therefore, to provide foundational information for determining the value proposition of early physical therapist management of neck pain. To address this purpose, we compared the odds of achieving a meaningful reduction in disability and pain and we compared the metrics of value and efficiency between patients receiving early physical therapist management and delayed physical therapist management to determine the impact of timing of physical therapist management. We hypothesized that patients who received physical therapist management earlier during an episode of neck pain would have the best odds of clinical improvement and the greatest value and efficiency for decreasing disability and pain.

## Methods

### Database

Data for this retrospective cohort analysis was extracted from the Rehabilitation Outcomes Management System (ROMS). This rehabilitation database contains demographics, patient characteristics, clinical outcomes and rehabilitation utilization data that is maintained by Intermountain Healthcare, a private, non-profit integrated healthcare system. The study protocol was approved by the Institutional Review Board of Intermountain Healthcare.

### Patients

**Fig. 1:**
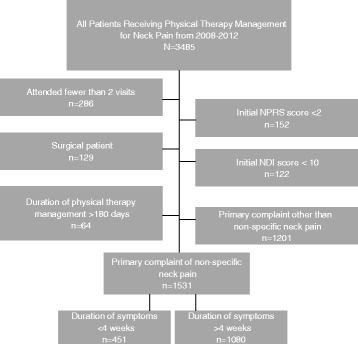
Derivation of the sample

Patients included in this study received physical therapist management for the primary complaint of neck pain between dates January 1, 2008 to December 31, 2012 from 13 outpatient physical therapy clinics located in Salt Lake City, Utah and surrounding regions. Inclusion criteria for these analyses were: non-surgical patient, Neck Disability Index (NDI) score of 10 or greater and Numerical Pain Rating Scale (NPRS) score of 2 or greater at initial evaluation, 2 or more visits, duration of physical therapist management of less than 180 days and have a self-reported primary complaint of non-specific neck pain. The inclusion criteria were implemented to permit the evaluation of clinical outcomes [24, 25] that exceed measurement error [26, 27]. See Fig. 1 for derivation of sample.

### Symptom duration

At initial evaluation, patients reported their primary complaint and neck pain symptom duration. This information was recorded by the physical therapist and entered into the ROMS database. Patients utilized in this analysis were identified in the ROMS database if they self-reported non-specific neck pain as their primary complaint. The patients were categorized as receiving “early physical therapist management” of neck pain if they reported non-specific neck pain with duration less than 4 weeks and “delayed physical therapist management” if they reported non-specific neck pain duration greater than 4 weeks. This categorization is supported in the low back pain literature where early physical therapist management has been defined as management within 2 weeks [10, 11] or within 4 weeks [28] of either self-report of duration of symptoms or the time from primary care provider consultation to physical therapy consultation. Therefore in this study the authors utilized a modest estimate of a cut-off of duration of symptoms of 4 weeks to define early management, to be inclusive of patient symptoms consistent with acute and early sub-acute pain stages [29].

### Clinical outcomes

The primary clinical outcome measures used in these analyses were the Neck Disability Index (NDI) and the Numerical Pain Rating Scale (NPRS). The NDI is a condition specific outcome measure that is a commonly used outcome measure for people with neck pain and is the most studied and well-established of all the outcome measures for neck pain [30] and it is found to be reliable and valid [30–34]. The NDI is comprised of 10 items; seven items related to activities of daily living, two items related to pain, and one item related to concentration [35]. Each item is scored from 0 to 5. The total score is expressed as a percentage and is reflective of a level of disability related to neck pain where high percentages are related to higher disability. In addition to the NDI, the NPRS is also a commonly used outcome measure for patients with neck pain [36]. The NPRS exhibits fair to moderate test-retest reliability in patients with mechanical neck pain and shows adequate responsiveness in this patient population [26, 36]. The NPRS is an 11 point scale, anchored with 0 rated as “no pain” and 10 rated as “worst pain” imaginable. Patients are asked to rate their current pain using this scale.

In order to determine whether patients experienced a meaningful improvement the Minimal Clinically Important Difference (MCID) was calculated for the NDI and NPRS. MCID values assist in determining the minimal amount of change that represents a clinically important difference for the patient. The most rigorous estimate of MCID for the NDI for patients with neck pain has been reported as a 19 percentage point change (9.5 raw score). Other estimates of MCID for patients with neck pain include a 10 percentage point change (5 raw score) [37] and 15 percentage point change (7.5 raw points) [35]. The authors used the MCID cut point of a 19 percentage point change because it is the most rigorous estimate and it exceeds the measurement of error for the NDI (10 percentage points) [33]. The MCID for the NPRS in patients with neck pain is 1.3 points [36] and this value was the cut point utilized for the MCID on the NPRS in this study.

Next, we wanted to determine the value proposition of early physical therapist management of neck pain by examining the metrics of value and efficiency. Value is defined as the change in outcome per dollar. In this study, we calculated two value metrics using the changes in NDI and on the NPRS during an episode of physical therapy. The metric of value for disability was calculated by dividing the change score on the NDI (Initial NDI score - NDI score at the last visit = change in NDI score) during an episode of care by the total charges for physical therapy multiplied by 100 during an episode of care ((Change in NDI/Charges for PT) x100)). This calculation allows for the interpretation of change in disability per 100 dollars. The metric of value for pain intensity was calculated by dividing the change score on the NPRS (Initial NPRS score - NPRS score at the last visit = change in NPRS score) during an episode of care by the total charges for physical therapy multiplied by 100 during an episode of care ((Change in NPRS/Charges for PT) x100)). Smaller numbers indicate lower value and larger numbers higher value.

A variable for efficiency of physical therapist management of neck pain for decreasing disability was also calculated and has been previously reported for musculoskeletal conditions [38]. The authors also wanted to calculate the efficiency of physical therapist management of neck pain for decreasing pain. Efficiency in decreasing disability is calculated by dividing the change in NDI score by the total number of visits during an episode of care (Change in NDI/# of visits to PT) [38]. Efficiency in decreasing pain is calculated by dividing the change in NPRS score by the total number of visits during an episode of care (Change in NPRS/# of visits to PT). Smaller values represent lower efficiency or less improvement per visit and larger values higher efficiency or more improvement in disability or pain per visit.

## Data analysis

Descriptive statistics and clinical characteristics were calculated for all patients in this sample. Means and standard deviations were reported for normally distributed continuous variables, median and interquartile range for number of visits and percentages were reported for categorical variables. Baseline patient characteristics, clinical outcomes and utilization of physical therapy were compared among timing groups using chi-squared analyses for categorical variables and independent t-tests for normally distributed continuous variables or Mann Whitney U tests for non-normally distributed continuous variables.

The main outcomes of interest in this study were the odds of patients achieving MCID on the NDI and NPRS and the metrics of value and efficiency. Model selection for all analyses were performed using an initial set of potentially confounding variables based on previous literature on prognostic factors for neck pain such as age and gender [39] and conceptually driven variables (baseline scores for pain and disability, duration of treatment and number of visits) [40]. Variables were removed if they did not improve model fit as measured by the explained variance in the exploratory models.

Both unadjusted and adjusted analyses were performed with separate binary logistic regression models to compare the odds of achieving MCID on the NDI and the NPRS between patients receiving early physical therapist management and delayed physical therapist management. Covariates in the initial adjusted models included age, gender, and duration of treatment, number of visits and baseline NDI and NPRS scores. All covariates were retained in the final model with the exception of baseline scores on the NDI in the model estimating MCID on the NPRS and baseline scores on the NPRS in the model estimating MCID on the NDI. Odds ratios with 95 % confidence intervals were reported.

Separate generalized linear regression models, normal distribution with identity link function, were estimated to compare the value for disability and value for pain between patients receiving early physical therapist management and delayed physical therapist management. Covariates in the initial models included age, gender, number of visits, duration of treatment and baseline scores for the NDI and NPRS. All covariates were retained in the final model when estimating value for disability and when estimating value for pain, with the exception of baseline scores on the NDI, all covariates were retained in the final model. Separate generalized linear regression models, normal distribution with identity link function, were estimated to compare the efficiency of physical therapist management for both change in disability and change in pain per visit between patients with receiving early physical therapist management and delayed physical therapist management. Covariates in the initial models included age, gender, duration of treatment and baseline scores for NDI and NPRS. In the final models estimating efficiency for change in pain and change in NDI the covariates included age, gender, admission NPRS score and duration of treatment and baseline score of outcome of interest. Estimates of means and 95 % confidence intervals between groups were reported. Data analysis was performed using SPSS statistical software (version 21.0). Significance level for all analyses was set at 0.05.

## Results

One thousand five hundred thirty-one patients with non-specific neck pain were identified from the database after exclusion criteria were met. See Fig. 1. Of these patients, 451 patients received early physical therapist management and 1080 patients received delayed physical therapist management. Patients receiving early physical therapist management overall were younger, had slightly more males and had greater percentages of patients achieving MCID on both the NPRS and the NDI. These patients also had slightly higher average charges per visit and for an episode of care but these were not significant differences compared to delayed physical therapist management. There was no difference in the median number of visits between groups. See Table 1.

**Table 1:** Descriptive and clinical characteristics of the sample

	Early physical therapist management (Duration of symptoms <4 Weeks)	Delayed physical therapist management (Duration of symptoms >4 Weeks)	*P*
	*n* = 451	*n* = 1080	
Age	46.23 (15.41)	52.41 (16.77)	0.07
Gender (% Female)	64.1 %	70.5 %	0.01
Initial NDI Score	40.18 (17.54)	34.31 (15.22)	<0.001
Discharge NDI Score	23.86 (17.34)	25.63 (16.31)	0.06
Initial NPRS Score	5.96 (2.23)	5.28 (2.14)	<0.001
Discharge NPRS Score	3.20 (2.50)	3.57 (2.37)	0.01
Percentage of Patients Achieving MCID on the NDI	38.8 %	23.5 %	<0.001
Percentage of Patients Achieving MCID on the NPRS	68.1 %	64.2 %	<0.001
Number of Physical Therapy Visits (Median, IQR)	5 (5)	5 (4)	0.82
Total Charges for an Episode of Physical Therapy ($USD)	$918.42 ($667.47)	$882.73 ($621.23)	0.29
Average Charges per Physical Therapy Visit	$162.56 (33.83)	$158.88 (40.07)	0.08

Abbreviations: *MCID* Minimal Clinically Important Difference, *NDI* Neck Disability Index, *NPRS* Numerical Pain Rating Scale, *IQR* Interquartile range

All values represent Mean (standard deviation) or percentages

The odds of achieving MCID or the amount change on an outcome measure that needs to occur for a patient to identify as important is reported in Table 2. As hypothesized, patients who received early physical therapist management demonstrated increased odds of achieving MCID on the NDI (aOR = 2.01, 95 % CI 1.57, 2.56) and MCID on the NPRS (aOR = 1.82, 95 % CI 1.42, 2.38) compared to patients who received delayed physical therapist management.

**Table 2:** Unadjusted and adjusted estimates of odds of achieving MCID

	Unadjusted Odds Ratio (OR)		Adjusted Odds Ratio (aOR)	
	Early physical therapist management (Duration of symptoms <4 Weeks)	Delayed physical therapist management (Duration of symptoms >4 Weeks)	Early physical therapist management (Duration of symptoms <4 Weeks)	Delayed physical therapist management (Duration of symptoms >4 Weeks)
Odds of achieving MCID NDI	2.06 (1.63, 2.61)	REF	2.01 (1.57, 2.56)	REF
Odds of achieving MCID NPRS	2.05 (1.63,2.59)	REF	1.82 (1.42, 2.38)	REF

Abbreviations: *MCID* Minimal Clinically Important Difference, *NDI* Neck Disability Index, *NPRS* Numerical Pain Rating Scale

Values represent odds ratio with 95 % Confidence Intervals

Patients who received early physical therapist management demonstrated better value in decreasing disability and pain compared to patients who received delayed physical therapist management. When controlling for covariates, patients receiving early physical therapist management demonstrated a 2.27 percentage point change (95 % CI 2.03, 2.51) in disability score per 100 dollars compared to patients who received delayed physical therapist management who demonstrated a 1.22 percentage point change in NDI per 100 dollars spent (95 % CI 1.06,1.38). Patients who received early physical therapist management also demonstrated a greater change in pain per 100 dollars (0.38 points, 95 % 0.34, 0.41) compared to patients who received delayed physical therapist management (0.28 points, 95 % CI 0.25, 0.30).

Patients who received early physical therapist management also experienced more efficiency in reducing disability and pain with physical therapist management of neck pain. The patients who received early physical therapist management achieved a 3.44 percentage point change in NDI score per visit (95 % CI 3.11, 3.78) and 0.57 point change (95 % CI 0.52, 0.62) in pain compared to a 1.81 percentage point change (95 % CI 1.58, 2.03) in NDI score and 0.42 point change (95 % CI 0.38, 0.45) in pain per visit when patients received delayed physical therapist management. Both adjusted and unadjusted mean estimates and 95 % CI are reported in Table 3.

**Table 3:** Unadjusted and Adjusted estimates of mean value and efficiency

	Unadjusted estimated eean		Adjusted estimated mean	
	Early physical therapist management (Duration of symptoms <4 Weeks)	Delayed physical therapist management (Duration of symptoms >4 Weeks)	Early physical therapist management (Duration of symptoms <4 Weeks)	Delayed physical therapist management (Duration of symptoms >4 Weeks)
Value-Disability (Change in NDI/100 dollars)	2.42 (2.16,2.68)	1.27 (1.12,1.41)	2.27 (2.03,2.51)	1.22 (1.06,1.38)
Value-Pain (Change in NPRS/100 dollars)	0.42 (0.38,0.46)	0.26 (0.24,0.28)	0.38 (0.34,0.41)	0.28 (0.25,0.30)
Efficiency-Disability (Change in NDI per Visit)	3.65 (3.27,4.03)	1.84 (1.64,2.05)	3.44 (3.11,3.78)	1.81 (1.58,2.03)
Efficiency-Pain (Change in Pain per visit)	0.64 (0.57,0.70)	0.39 (0.35,0.42)	0.57 (0.52,0.62)	0.42 (0.38,0.45)

Abbreviations: *NDI* Neck Disability Index, *NPRS* Numerical Pain Rating Scale

Values represent mean estimates with 95 % Confidence Intervals

## Discussion

Improving value in musculoskeletal healthcare has emerged as an important objective and the Institute of Medicine (IOM) has made the recommendation that the public at large and people with pain, in particular, would benefit from a better understanding of pain and its treatment, in order to encourage timely care and improve medical management [41]. Moreover the US healthcare system, which currently operates in FFS model, has contributed to an uncontrollable rise in healthcare costs [1–3]. This threatens the long-term performance of the healthcare delivery system and the rise in healthcare costs is widely viewed to be unsustainable economically [6]. Payment models are evolving from FFS models to other payment strategies that include incentives for quality, outcomes, improved patient experience, and reduced costs; essentially paying for value, not volume [42]. Therefore, there has been a call to action to determine the value proposition of care provided by healthcare providers, such as physical therapists, for management of musculoskeletal pain [6]. Value proposition can be operationalized as demonstrating a reduction in disability, pain and improvement in health status of individuals through more cost-effective management of neck pain [6]. The results of our study describe part of the value proposition for early physical therapist management of neck pain. In this cohort we determined that when neck pain is managed early, within the first four weeks of symptoms, the patient is more likely to have a meaningful reduction in disability and pain, will demonstrate greater value per dollar for physical therapy and experience more efficient care when compared to patients who received delayed management or when symptoms were present for greater than four weeks. Consistent with the key components of a value proposition, these results can be interpreted from the perspectives of three primary stakeholders; the patient, the payer and the provider.

The indirect costs for patients with neck pain can potentially supersede the direct costs to the healthcare system. A study in the Netherlands found that direct costs, such as healthcare, amounted to just 23 % of this figure while indirect costs, such as work absenteeism and disability, amounted to 77 % of the total costs [43]. In this study, when patients received early physical therapist management they were twice as likely to actually experience a meaningful change in disability and were 1.8 times as likely to achieve a meaningful reduction in pain in comparison if they waited longer than 4 weeks to receive care. This finding supports that early management of neck pain has the potential to decrease the indirect costs associated with neck pain and improve the patient experience by decreasing disability, which can be significant [44], in patients with neck pain. But further research is needed to examine the relationship between early physical therapist management and indirect costs associated with neck pain through formal economic evaluation.

When investigating the value of early physical therapist management of neck pain from the payer perspective, our findings are consistent with the literature which reports that earlier physical therapist management of spine pain can lead to less resource utilization [10, 39, 45]. If examining charges alone in this cohort, although not significant, the patients receiving early physical therapist management incurred slightly higher charges for an episode of PT. But the value is realized when considering the decrease in disability and pain per dollars spent. Research supports that the level of disability at discharge is associated with both the short and long term prognosis for neck pain [45, 46]. Therefore it is cost beneficial for the payer to have a patient demonstrate a greater decrease in disability and pain in the short term with a few more dollars spent, then to increase the likelihood of recurrence [36] with a potential for increased downstream utilization of healthcare resources long term.

From the provider perspective, this study supports that the most efficient care can be provided when a patient is seen within the first four weeks of symptoms in order to experience the greatest decreases in their disability and pain. Bridging these findings with the patient perspective, this means it takes approximately half the number of visits when patients receive early physical therapist management to achieve a meaningful reduction (ie MCID) on the NDI and NPRS compared to patients who receive delayed management to achieve the same decrease in disability on the NDI and NPRS. Some may argue that patients receiving early physical therapist management of neck pain, may have different care seeking behavior due to experiencing more severe symptoms or simply this group experienced a regression towards the mean that accounted for the greater change in score compared to delayed physical therapist management. Although patients who received early physical therapist management had higher baseline NDI scores, the difference in the NDI scores between groups did not exceed error [36] nor were the groups categorized in different functional categories by their scores, i.e both categorized as having “moderate disability” on the NDI [47]. Early physical therapist management of neck pain, if considered as a value driven management approach has the potential to decrease the number of visits needed for physical therapists to effectively manage neck pain, but can also lead to patients experiencing meaningful reductions in pain and disability over a shorter period of time. Further research is needed to weigh the potential benefit of early physical therapist management against over treatment in patients with neck pain.

There are strengths in the current study. The concept of value proposition of early physical therapist management of neck pain was discussed from three perspectives: the patient, payer and provider. The benefit of early physical therapist management was potentially realized from all stakeholders. The policy implications of these findings provide support for early physical therapist management of neck pain. An additional strength of the study was the introduction of the metric of value specifically related to physical therapist management of neck pain. The metric of value calculated in this study is consistent with the definition of value in healthcare services, where value is defined the health outcomes achieved per dollar [23]. We examined the value of physical therapist management of neck pain utilizing the clinical outcomes of disability and pain. Interpreting these findings provides a meaningful way to communicate to stakeholders what the “return investment” for early physical therapist management of neck pain may be for systems that provide pathways for early management.

There are also limitations in the current study. The authors discuss the value of early physical therapist management of neck pain, not value of early physical therapist management of neck pain and its effect on downstream healthcare costs and utilization. Although this is a limitation, recent research in low back pain supports that providing early physical therapist management after consultation with a primary care provider was not associated with an increase in costs or utilization of specific services [48]. Therefore the authors feel that the potential benefit of early physical therapist management exceeds the risk of unnecessary or inefficient use of healthcare services. Moreover, this study only examined the effect of timing on outcomes and did not dissect the differences in actual treatments received by patients in each group. The authors were also limited to using charges for physical therapy rather than costs which are known to differ. Though there is the potential that the costs may be lower, the interpretation of the findings remains the same, which is the ratio of change in outcome measure over dollars. Lastly, this study was a retrospective cohort analysis where there is the potential for bias due to study design. Research suggests that are factors such as recurrence [49], severity [44], treatment preferences [50, 51] and psychosocial factors such as depressive symptoms [52] catastrophizing [53] and fear avoidance beliefs [54] that can adversely affect outcomes in patients with spine pain. These factors, which were unmeasured in this study could have introduced bias in patient grouping and difference in outcomes between groups. The authors acknowledge this potential for bias and attempted to minimize this bias with the inclusion of known prognostic factors and baseline pain and disability status where appropriate from the available variables.

Further research in needed to examine the effect of early physical therapist management of neck pain relative to downstream costs and utilizations and to make specific recommendations on interventional strategies based on timing of physical therapist management of neck pain.

## Conclusions

Policy makers and payers contribute to the value equation by designing health policies that promote access and use of timely, appropriate healthcare services [6]. In this study we provided evidentiary support for early management of neck pain where the patient, payer and provider may benefit. The implication of these findings suggest that healthcare systems that provide pathways for patients to receive early physical therapist management of neck pain may realize improved patient outcomes, increased efficiency in delivery of care and greater value. Further research is needed to determine the overall impact of promoting early physical therapist management of neck pain on the entire healthcare experience to elucidate the benefit relative to other management pathways.

## Abbreviations

aOR, Adjusted odds ratio; FFS, Fee for service; IOM, Institute of Medicine; MCID, Minimal Clinically Important Difference; NDI, Neck disability index; NPRS, Numerical Pain Rating Scale; NSNP, Non-specific neck pain; OR, Odds Ratio; PT, Physical Therapy; USD, United States Dollar.
